# How to Make a Creamy, Tasty Vegan Camembert

**DOI:** 10.1021/acscentsci.6c00840

**Published:** 2026-05-26

**Authors:** Marta Zaraska

## Abstract

Coaxing dairy-like textures and flavors from plant-based materials is a challenge for makers of vegan cheese.

## Graphical abstract

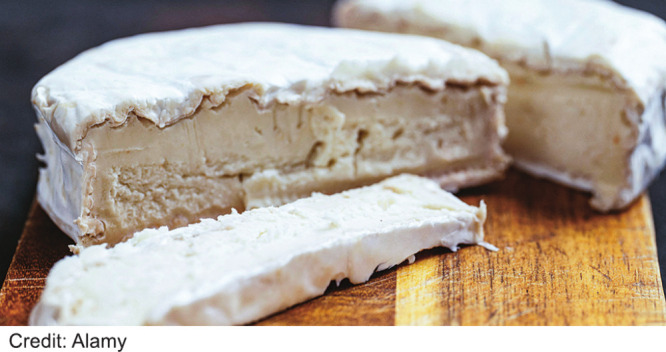

Inside a narrow, modern room, several large fridges with glass doors line the walls. Each fridge is filled with white, velvety wheels of what looks like Camembert in various stages of maturation. On the floor are bags of cashews. There are no dairy products in sight.

**Figure:**
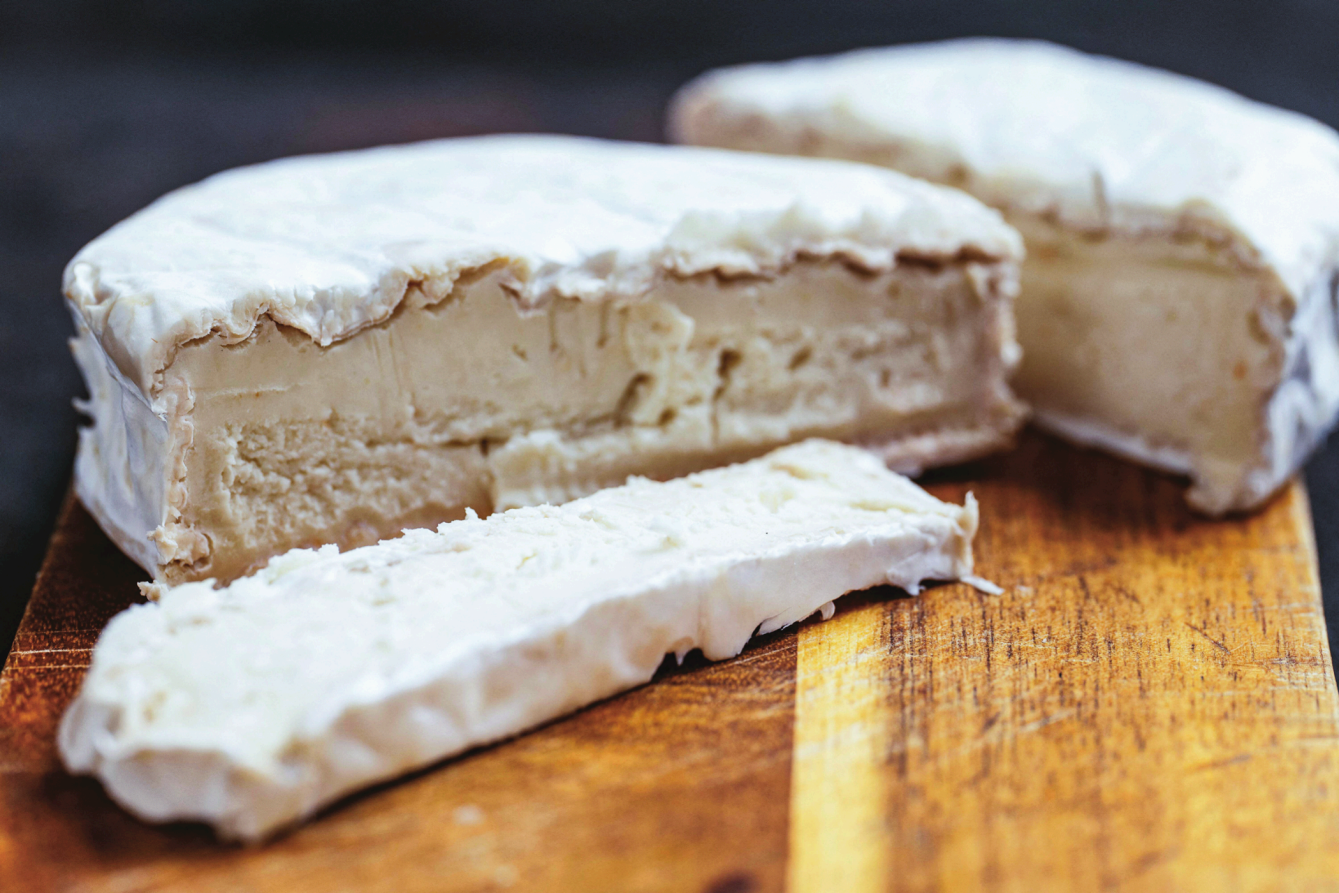
Vegan Camembert made from fermented cauliflower. Credit: Alamy.

This space in northern France is one of several across the country where a vegan version of mold-ripened Camembert is made. Replicating the flavors and textures of dairy cheese with plant fats and proteins is certainly not easy, but it is a puzzle that several groups of researchers are trying to solve.

Europe has the highest market share in vegan cheese. According to Alejandro Marangoni, a food scientist at the University of Guelph, plant-based mold-ripened cheeses offer potentially healthier alternatives to the highly processed cheddar-style blocks of vegan cheese, which are often just “coconut-oil-filled starch.”

Commercial vegan cheese first appeared on supermarket shelves in the 1980s, but on a molecular level, this cheese had little in common with dairy. That situation is still often the case. “It’s not healthy for you, it actually has no protein, it has a lot of fat, it has a lot of salt,” Marangoni says.

## The secrets of cheese making with milk

Dairy milk is mostly water, with tiny fat droplets dispersed in it alongside casein micelles—spherical aggregates of casein proteins. To make cheese from that suspension, cheese makers first add a starter culture, such as lactic acid bacteria, to the milk. As the pH drops, the caseins stick together in a network, trapping the fat: the milk curdles. Many traditional cheeses also use rennet, an enzyme that helps form stronger protein networks for firmer curds. Once a cheese maker drains the curds from the remaining liquid, whey, they end up with a fresh cheese (think the cottage kind).

To make an aged cheese, the curds are pressed, salted, and left so that the protein network increases its density, and enzymes work to develop flavor and texture. And when cheese makers add *Penicillium* molds to the curd, they can produce different styles of cheese depending on how the process is handled. In soft cheeses, such as Camembert, the mold grows on the surface, where it has access to oxygen. In blue cheeses, such as Roquefort, cheese makers pierce the maturing cheese to allow oxygen inside, and the mold develops within.

In cheeses where the mold grows on the surface, the enzymes that the mold produces diffuse inward. “They migrate into the interior of the product, and from the outside to the inside, the cheese begins to ripen,” says Łukasz Łopusiewicz, a microbiologist at the University of Greifswald and Vizja University. “This is where protein breakdown occurs as well as lipolysis of fats, and, as a result, this creamy center develops.”

**Figure:**
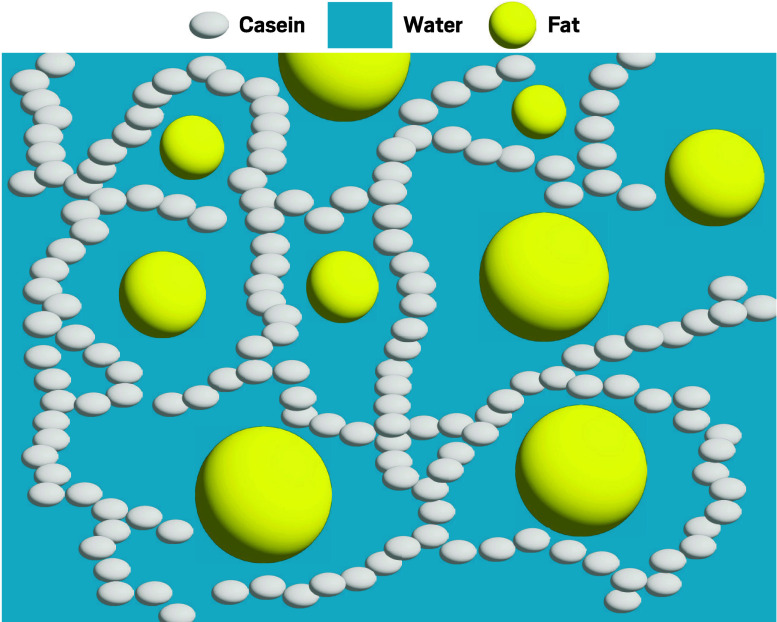
**Captured in casein:** In milk, casein forms micelles. In cheese, these micelles form a gel that gives cheese its soft and springy qualities. Replicating this gel with vegan protein sources is a challenge. Credit: Adapted from *J. Dairy Sci* .

## The challenges of vegan versions of cheese

Łopusiewicz spends many weeks turning over Camemberts, mopping up condensation, and checking if the velvety ovals ripen properly. He has aged dairy cheeses in his lab but also vegan versions made from potato, rapeseed, and flaxseed. While he admits that making vegan Camembert is a lot of work, he says what fascinates him is that *Penicillium* molds are able to grow on plant material to begin with. “From a molecular, biochemical, and genetic perspective, it’s extremely interesting,” he says. After all, plant proteins and fats are very much unlike those found in milk.

The top difference lies with the proteins. In milk, the caseins assemble into larger particles, and “cheese is basically a network of these particles,” says Elke Scholten, a food scientist at Wageningen University and Research. This network of casein micelles is responsible for the properties of dairy products. In a newborn mammal’s stomach, casein coagulates into curds, slowing digestion and making it easier for the baby to absorb nutrients. In cheese making, casein micelles give cheese its texture.

So far, food scientists haven’t found any plant proteins that behave the way caseins do. Biologically, plant proteins don’t need to, since they are not designed to slow digestion in newborn animals. Plant proteins not only don’t form micelles but are also “very insoluble,” Marangoni says. Without good water binding and flexibility, it’s hard to get that creaminess, that smoothness you experience in your mouth when you eat dairy cheese. You get grittiness instead-— a common complaint of vegan-cheese consumers.

Melting is an issue, too. In general, when dairy cheese is heated, the casein protein network loosens, yet plant proteins tend to “go the opposite direction,” Scholten says, with the network strengthening under heat.

To get the color and texture somewhat right, vegan-cheese makers often use a starch-based matrix filled with saturated fatty acids, then spiked with preservatives and colorants. To get a good melt, you can balance starch with plant protein and lipids. Scholten and her colleagues managed to do this in their 2023 experiments with mixtures of pea protein and sunflower oil. But plant fats also try the skills of vegan-cheese makers. Saturated fatty acids, which are particularly abundant in dairy cheese, tend to be solid at room temperature, but on a warm human tongue, they melt. According to Scholten, it’s that melting that we perceive as creaminess. Many plant oils contain larger amounts of unsaturated fatty acids and are liquid at room temperature, and even those that are solid, such as coconut and palm oils, don’t melt on the tongue in the same way that dairy fats do, she says.

## Adding a fermented boost to vegan cheeses

Fermenting plant-based cheeses might be an unexpected way to counteract the problems of vegan cheese, according to some researchers. “Exploiting the natural properties of fermentation can open up a lot of things,” Marangoni says. For one, fermentation can increase protein solubility and improve texture without the need for additives, he says. And two, it can boost flavor: in one 2023 study, fermenting pea protein not only improved the texture of the resulting cheese but also removed beany, off flavors.

If you take things further and mold ripen vegan cheese, chances are you will end up with even more creaminess, even better taste, and a delicious aroma. In 2020, Łopusiewicz and his colleagues fermented and mold ripened flaxseed-based cheese alternative using the common cheese mold *Penicillium camemberti* and a yeast, *Geotrichum candidum* together with lactic acid bacteria. Over a month of ripening, enzymes produced by the microorganisms broke down flaxseed proteins and cell-wall polysaccharides, releasing free amino acids and polyphenols. The texture also changed as the peptide bonds were broken and rearranged.

Next, Łopusiewicz and his colleagues experimented with lupine seeds. Over 49 days of ripening, *Penicillium camemberti* broke down the lupine protein even better than it did flaxseed protein, increasing the free amino acid content (although, admittedly, not as much and not as evenly as it does in dairy cheese). The researchers ended up with what looked like small, round carrot cakes with sugar icing on top, which had “a very intense mushroom-like aroma and taste,” Łopusiewicz says. In fact, he admits, the flavor might be too intense even for his liking. The texture, though, was very Camembert-like, he says.

Other groups of researchers have also shown that mold ripening can produce good Camembert analogs from various plant materials. For a 2025 study, researchers mixed faba-bean milk with coconut and rapeseed oils, which they then ripened with *Penicillium camemberti.* They showed that higher fat content made for a smoother, creamier cheese.

For a 2024 study, researchers aged eight different types of plant-based cheese. The cashew one looked the palest, the most Camembert-like; the pea one was green inside; and the hemp one was almost black. A pistachio cheese produced a particularly thick and even skin of velvety mold. One common problem, however, was aroma. When a panel of volunteers evaluated the smell of the vegan Camembert analogs on a scale of 0 to 9, most notes fell somewhere between 3 and 5.

What your nose may perceive as the unique smell of ripened cheese relies on methyl ketones such as 2-heptanone and 2-nonanone, which are produced when molds break down medium-chain saturated fatty acids in milk. In December, a study showed that in soy milk, neither *Penicillium camemberti* nor *Penicillium roqueforti* (a fungus typically used for making blue cheese) managed to produce the cheese-aroma-evoking ketones—hardly surprising considering that soy milk doesn’t contain the necessary precursors. The researchers experimented with adding various plant oils to the milk and discovered that only coconut oil enabled the molds to whip up cheese-like aromas. A mold-ripened soy-coconut Camembert did have similar levels of 2-heptanone to dairy cheese, yet overall, the scientists report that its aroma was still quite distinct.

The question is whether producers should even strive to replicate dairy cheese with plant-based materials; perhaps it’s good enough to have vegan cheese that’s different yet tasty in its own right. In one 2025 survey, more than 61% of customers said they’d welcome products that tasted good but didn’t mimic the taste of dairy cheese.

For Marangoni, making a great vegan cheese is not simply about combining the right types of protein with the right types of fat in experimentally tested proportions. At some point, he says, “the science stops and the art starts.” And if the result is not an exact copy of dairy, yet healthy and delicious, he is fine with that, too.

*Marta Zaraska is a freelance contributor to* Chemical & Engineering News, *an independent news publication of the American Chemical Society.* A version of this story appeared in C&EN.

